# Association between serum hemoglobin and major cardiovascular adverse event in Chinese patients with ST‐segment elevation myocardial infarction after percutaneous coronary intervention

**DOI:** 10.1002/jcla.24126

**Published:** 2021-12-10

**Authors:** Yulu Yang, Yun Huang

**Affiliations:** ^1^ Department of Geriatrics Union Hospital Tongji Medical College Huazhong University of Science and Technology 1277 JieFang Avenue Wuhan Hubei 430022 China

**Keywords:** hemoglobin, major adverse cardiovascular event, myocardial infarction, percutaneous coronary intervention, prognosis

## Abstract

**Background:**

ST‐segment elevation myocardial infarction (STEMI) is a common clinical acute and severe disease, and it is of great significance to evaluate the prognosis of these patients. Hemoglobin levels are associated with a variety of diseases, but studies on Chinese patients with STEMI after percutaneous coronary intervention (PCI) have not been sufficient.

**Methods:**

This was a secondary analysis based on a prospective cohort study of patients undergoing PCI in Taizhou, Zhejiang, China. We performed multivariable logistic regression to explore the association between the serum hemoglobin and the incidence of major cardiovascular adverse event (MACE) in patients after PCI. We also used a generalized additive model and smooth curve fitting to explain the nonlinear relationship after adjusting the potential confounders. Finally, the heterogeneity among specific groups was examined by subgroup analysis.

**Results:**

Of all 462 patients enrolled in this study, 118 (25.54%) developed MACE. There was a negative correlation between serum hemoglobin and MACE in all three models (hazard ratio [HR] 0.82, 95% confidence interval [CI 0.72, 0.93], HR 0.86, 95% CI [0.76,0.98], and HR 0.87, 95% CI [0.74,0.98], respectively). In the subgroup analysis, the negative correlation existed between the patients who had myocardial infarction (MI) history (*p* for interaction = 0.0059) after adjusting covariates. However, no significant differences were found between age and sex groups (*p* for interaction = 0.1381, 0.4103, respectively).

**Conclusion:**

Our results indicated that patients who received PCI with low preoperative hemoglobin were more likely to develop MACE, especially if they have already had a history of MI.

## Graphical abstract

The multivariate logistic regression showed a negative association between baseline hemoglobin and MACE in STEMI patients after PCI. In addition, patients in the highest tertile had a 50% lower chance of developing MACE than those in the lowest tertile.

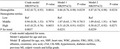

## 1 INTRODUCTION

ST‐segment elevation myocardial infarction (STEMI) is characterized by persistent chest pain, elevated myocardial injury markers, and ST‐segment elevation in at least two consecutive leads on electrocardiogram. Worldwide, STEMI is the most common single cause of death, causing an enormous medical burden, while its incidence is still on the rise.^1^ With the development of interventional technology, percutaneous coronary intervention (PCI) has brought great clinical benefits to more and more patients with STEMI.^2^ At the same time, the study of its complications and risk factors has gained more attention from researchers.^3^, ^4^ Previous studies have shown that preprocedural hemoglobin levels are associated with adverse clinical events after PCI, such as in‐hospital complications,^5^ higher bleeding risk,^6^ and poor long‐term outcomes.^7^ However, these studies had evident regional diversity, and the researches on the Chinese population were still insufficient. In the present study, we selected Chinese people to investigate the relationship between baseline hemoglobin level and major cardiovascular adverse event (MACE) in patients with STEMI after PCI.

## 2 METHODS

### 2.1 Data source and study population

Raw data were uploaded by Yang et al. to the “DATA‐DRYAD” website (https://datadryad.org/). Yang et al. have authorized the ownership of the original data to the data‐dryad website. Therefore, we utilized these data for secondary analysis on a different hypothesis without infringing the authors’ rights.

This was a prospective cohort study conducted at Taizhou, Zhejiang, China from January 2010 to October 2014. The Ethics Committee of the First People's Hospital of Taizhou approved the study. It enrolled 464 STEMI patients admitted to the hospital within 12 h of onset and underwent PCI. All patients were followed up for 30 months after PCI to collect their prognostic data.^8^ Two participants with missing serum hemoglobin data were excluded and the remaining 462 entered the final analysis.

### 2.2 Variables

In the initial observation cohort, the clinical records of patients receiving PCI were derived from their clinical information system and stored in their data repository and analysis system. We obtained the variables from the database. In the present study, serum hemoglobin concentration was used as the independent variable, and the dependent variable was whether MACE occurred. MACE was defined as a series of events, including cardiac death, recurrent myocardial infarction (MI) of rented target vessel, cardiogenic shock, and congestive heart failure. The covariates were as follows: age, sex, systolic blood pressure (SBP), heart rate, white blood cells (WBC), platelet, fasting plasma glucose (FBG), high‐density lipoprotein‐cholesterol (HDL), low‐density lipoprotein‐cholesterol (LDL), albumin, creatinine, uric acid, cardiac troponin I (cTnI), creatine kinase MB (CK‐MB), hypertension, diabetes mellitus, previous MI, culprit vessels, and killip grade.

### 2.3 Statistical analysis

All statistical analyses were performed using package R, version3.4.3 (http://www.r‐project.org), and EmpowerStats software (http://empowerstats.com). *p* < 0.05 was considered statistically significant. Continuous variables were presented as mean ± standard deviation or median (maximum, minimum), while categorical variables were reported as a percentage. For continuous variables, a linear regression model was used to calculate the differences between groups. For categorical variables, the Chi‐square test was used for analysis. Multivariable logistic regression models were performed to explore the relationship between the independent variable serum hemoglobin and MACE occurrence in patients after PCI. Generalized additive model and smooth curve fitting were tried to explain the nonlinear relationships after adjusting the same covariates. Then, subgroup analyses were used to find the heterogeneity between different groups stratified by age, sex, hypertension, diabetes mellitus, previous MI, albumin, creatinine, killip grade, and culprit vessels.

## 3 RESULTS

### 3.1 Baseline characteristics of participants

The description of basic clinical characteristics, laboratory examinations, and angiographic results are shown in Table 1. Of 462 patients enrolled in the study, 118 developed MACE, and 344 did not during follow‐up. 76.62% of the participants are male, and the mean age is 62.97 ± 11.92. Among different groups of age, heart rate, hemoglobin, creatinine, cTnI, and killip grade, MACE occurrence is significantly different, whereas others are not significantly different.

**TABLE 1:** Baseline characteristics of patients (n = 462)

Variable	Total	No MACE	MACE	*p*‐value
N	462	344	118	
Age (years, mean ± SD)	62.97 ± 11.92	61.60 ± 11.54	66.95 ± 12.16	<0.001
Sex, n (%)				0.172
Male	354 (76.62)	269 (78.20)	85 (72.03)	
Female	108 (23.38)	75 (21.80)	33 (27.97)	
SBP (mmHg, mean ± SD)	131.83 ± 27.15	131.70 ± 27.37	132.22 ± 26.59	0.858
Heart rate (/min, mean ± SD)	76.96 ± 17.13	76.09 ± 16.58	79.48 ± 18.51	0.035
Hemoglobin (g/dl, mean ± SD)	14.38 ± 1.72	14.53 ± 1.71	13.94 ± 1.67	0.001
WBC (×10^9^/L, mean ± SD)	10.08 ± 3.64	9.90 ± 3.55	10.60 ± 3.86	0.072
Platelet (×10^9^/L, mean ± SD)	231.97 ± 56.25	229.15 ± 54.68	240.18 ± 60.08	0.066
FBG (mmol/L, mean ± SD)	7.67 ± 2.53	7.66 ± 2.48	7.67 ± 2.68	0.977
HDL (mmol/L, mean ± SD)	1.20 ± 0.27	1.19 ± 0.27	1.25 ± 0.28	0.062
LDL (mmol/L, mean ± SD)	3.04 ± 0.72	3.03 ± 0.73	3.07 ± 0.72	0.605
Albumin (g/L, mean ± SD)	37.94 ± 3.84	37.97 ± 3.82	37.88 ± 3.91	0.827
Creatinine (mmol/L, mean ± SD)	74.64 ± 22.75	74.07 ± 24.71	76.28 ± 15.64	0.011
Uric acid (mmol/L, mean ± SD)	337.11 ± 73.72	338.40 ± 71.27	333.34 ± 80.66	0.520
cTnI [ng/mL, median (min–max)]	13.55 (0.02–43.20)	12.60 (0.02–43.20)	21.45(0.02–39.10)	0.015
CK‐MB [U/L, median (min–max)]	106.00 (9.00–451.00)	104.00 (9.00–451.00)	131.50 (13.00–357.00)	0.429
Hypertension, n (%)	264 (57.14)	192 (55.81)	72 (61.02)	0.324
Diabetes mellitus, n (%)	149 (32.25)	108 (31.40)	41 (34.75)	0.502
Previous MI, n (%)	55 (11.90)	36 (10.47)	19 (16.10)	0.103
Culprit vessels, n (%)				0.526
LAD	231 (50.00)	167 (48.55)	64 (54.24)	
LCX	72 (15.58)	54 (15.70)	18 (15.25)	
RCA	159 (34.42)	123 (35.76)	36 (30.51)	
Killip grade n (%)				0.002
I	350 (75.76)	273 (79.36)	77 (65.25)	
>I	112 (24.24)	71 (20.64)	41 (34.75)	

Note

Mean ± SD or median (max, min) for continuous variables; *p*‐value was calculated by linear regression model.

% for categorical variables; *p*‐value was calculated by Chi‐square test.

Abbreviations: CK‐MB, creatine kinase MB; cTnI, cardiac troponin I; FBG, fasting plasma glucose; HDL, high‐density lipoprotein‐cholesterol; LAD, left anterior descending; LCX, left circumflex; LDL, low‐density lipoprotein‐cholesterol; MACE, major cardiovascular adverse event; MI, myocardial infarction; RCA, right coronary artery; SBP, systolic blood pressure; SD, standard deviation; WBC, white blood cells.

### 3.2 The association of serum hemoglobin with MACE in patients after PCI

Three multivariate logistic regression models were constructed to investigate the relationship between serum hemoglobin and MACE (Table 2): Crude model, without covariate adjustment; Model 1, adjusting for age, sex; Model 2 was adjusted for age, sex, SBP, heart rate, WBC, platelet, FBG, HDL, LDL, albumin, creatinine, uric acid, cTnI, CK‐MB, hypertension, diabetes mellitus, previous MI, culprit vessels, and killip grade. In the crude model, serum hemoglobin was negatively associated with MACE (hazard ratio [HR] 0.82, 95% confidence interval [CI 0.72, 0.93], *p* = 0.0016). After adjusting for confounders, this negative association remained in Model 1 (HR 0.86, 95% CI [0.76, 0.98], *p* = 0.0239) and Model 2 (HR 0.87, 95% CI [0.74, 0.98], *p* = 0.0234). When serum hemoglobin was converted from a continuous variable to a categorical variable (tertile), the trend test remained significant (*p* = 0.0259). Meanwhile, patients in the highest tertile had a 50% lower chance of developing MACE than those in the lowest tertile.

**TABLE 2:** Relationship between hemoglobin and MACE

	Crude model HR (95% CI)	*p*‐value	Model 1 HR (95% CI)	*p*‐value	Model 2 HR (95% CI)	*p*‐value
Hemoglobin	0.82 (0.72, 0.93)	0.0016	0.86 (0.76, 0.98)	0.0239	0.87 (0.74, 0.98)	0.0234
Hemoglobin tertiles						
Low	Ref		Ref		Ref	
Middle	0.94 (0.58, 1.53)	0.7974	1.07 (0.65, 1.79)	0.7813	1.09 (0.62, 1.91)	0.7601
High	0.43 (0.25, 0.76)	0.0033	0.51 (0.29, 0.91)	0.0231	0.50 (0.27, 0.92)	0.0271
*p* for trend	0.0037		0.0251		0.0259	

Note

Crude model adjusted for none.

Model I adjusted for age and sex.

Model Ⅱ adjusted for age, sex, SBP, heart rate, WBC, platelet, FBG, HDL, LDL, albumin, creatinine, uric acid, cTnI, CK‐MB, hypertension, diabetes mellitus, previous MI, culprit vessels, and killip grade.

Abbreviations: CI, confidence interval; CK‐MB, creatine kinase MB; cTnI, cardiac troponin I; FBG, fasting plasma glucose; HDL, high‐density lipoprotein‐cholesterol; HR, hazard ratio; LDL, low‐density lipoprotein‐cholesterol; MACE, major cardiovascular adverse event; MI, myocardial infarction; SBP, systolic blood pressure; WBC, white blood cells.

**FIGURE 1:**
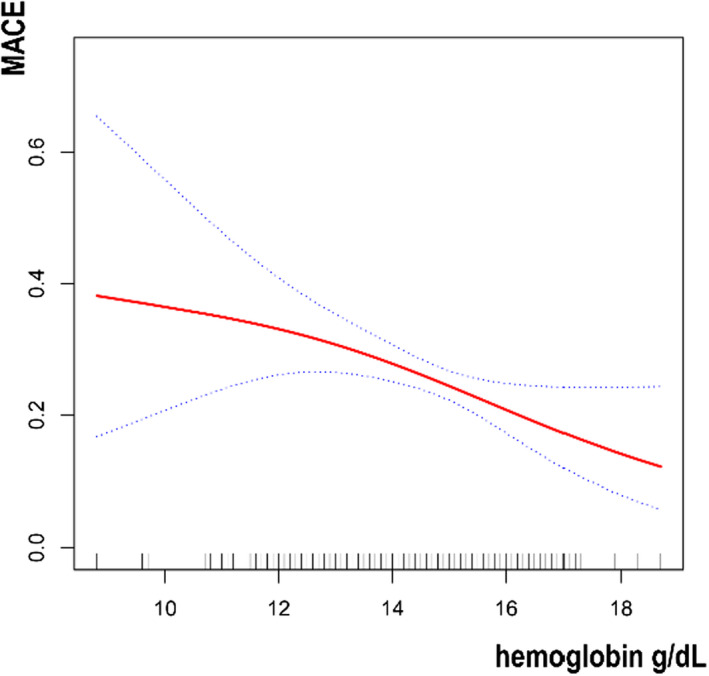
Association between hemoglobin and MACE in the whole population. MACE, major adverse cardiovascular event

**FIGURE 2:**
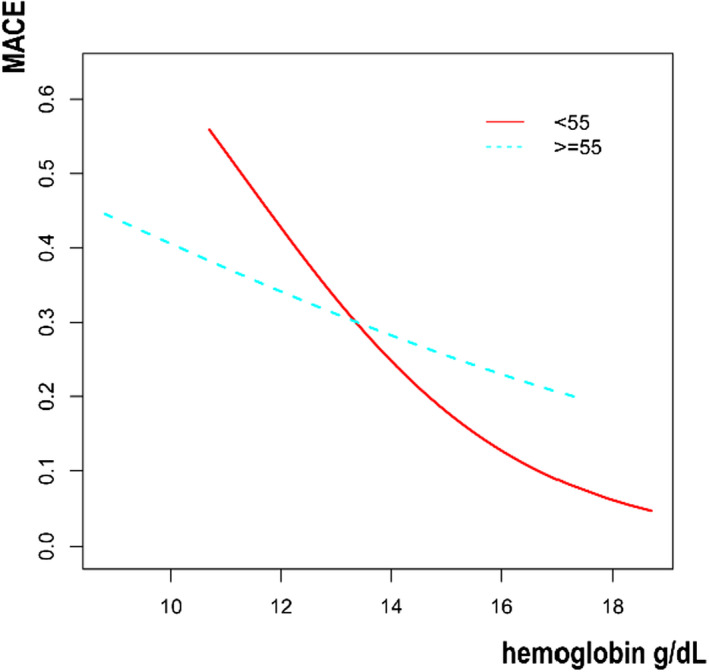
Association between hemoglobin and MACE stratified by age. MACE, major adverse cardiovascular event

**FIGURE 3:**
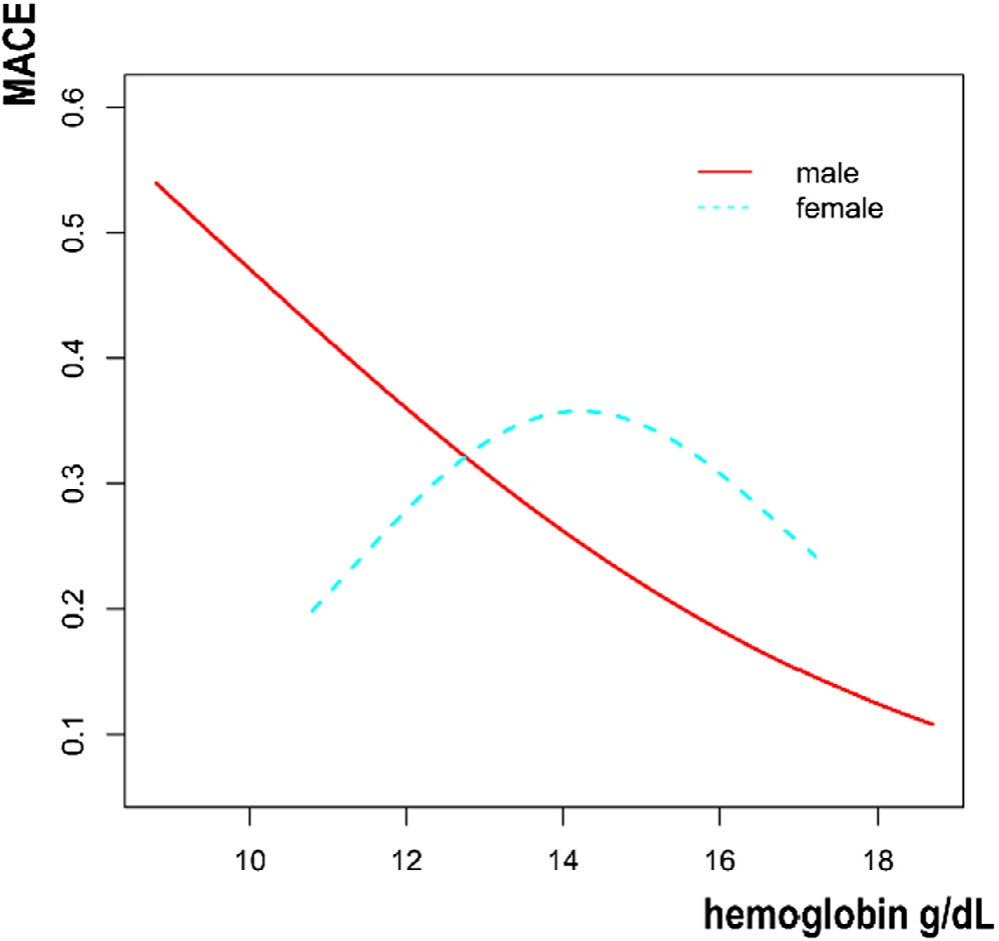
Association between hemoglobin and MACE stratified by sex. MACE, major adverse cardiovascular event

Then, we tried to use generalized additive models and smooth curve fittings to find the visual relationship between hemoglobin and MACE stratified by age and sex, respectively (Figures 1, 2, 3).

We also performed subgroup analyses stratified by age, sex, hypertension, diabetes mellitus, previous MI, albumin, creatinine, killip grade, and culprit vessels, and results are shown in Table 3. The test for interactions was significant for previous MI (*p* for interaction = 0.0059). We also observed that the negative association between hemoglobin and MACE occurrence was more significant in patients who have had a history of MI (HR = 0.32, 95% CI = 0.14, 0.73). However, the test for interactions was not statistically significant for age, sex, hypertension, diabetes mellitus, albumin, creatinine, killip grade, and culprit vessels (*p* values for interactions were larger than 0.05).

**TABLE 3:** Subgroup analysis stratified by age, sex, hypertension, diabetes mellitus, previous MI, albumin, creatinine, killip grade, and culprit vessels

	n	HR	95% CI low	95% CI high	*p*‐value	*p* (interaction)
Age						0.1381
<55	107	0.67	0.48	0.93	0.0155	
≥55	353	0.87	0.75	1.01	0.0685	
Sex						0.4103
Male	353	0.83	0.70	0.98	0.0319	
Female	107	0.98	0.69	1.39	0.9046	
Hypertension						0.6398
No	196	0.88	0.72	1.08	0.2298	
Yes	264	0.82	0.68	1.00	0.0452	
Diabetes mellitus						0.5297
No	312	0.83	0.70	0.98	0.0244	
Yes	148	0.92	0.70	1.20	0.5235	
Previous MI						0.0059
No	55	0.87	0.75	1.01	0.0742	
Yes	405	0.32	0.14	0.73	0.0071	
Albumin						0.0832
Low	216	0.71	0.56	0.89	0.0035	
High	244	0.92	0.77	1.11	0.3975	
Creatinine						0.2048
Low	225	0.76	0.60	0.96	0.0193	
High	235	0.91	0.76	1.09	0.3195	
Killip grade						0.5691
I	348	0.85	0.72	1.01	0.0647	
>I	112	0.93	0.72	1.19	0.5512	
Culprit vessels						0.4786
LAD	230	0.76	0.62	0.92	0.0059	
LCX	72	1.04	0.57	1.91	0.9006	
RCA	158	0.89	0.67	1.17	0.3998	

Abbreviations: CI, confidence interval; HR, hazard ratio; LAD, left anterior descending; LCX, left circumflex; MI, myocardial infarction; RCA, right coronary artery.

## 4 DISCUSSION

In the present study, 462 patients undergoing PCI were included. The results revealed a significant negative association between serum hemoglobin and MACE occurrence. For every 1 g/dl increase in hemoglobin, the incidence of MACE decreased by 13%. Additionally, with the increase of hemoglobin, the incidence further decreased in the participants who had MI history. Furthermore, we also used Figures 1, 2, 3 to show the relationship between hemoglobin and MACE in different ages and genders, but further stratified analysis found no significant difference.

Recent studies have reported the relationship between preoperative hemoglobin and adverse outcomes after PCI, which is consistent with the result of the present study. Leonardi et al. grouped the patients with acute coronary syndrome according to the severity of hemoglobin decline and found that both mild and severe hemoglobin decline was associated with increased 1‐year mortality regardless of whether bleeding occurred.^9^ A recent Mayo Clinic study found that the presence or absence of anemia was an independent predictor of all‐cause and non‐cardiac mortality after PCI in patients with acute coronary syndrome, according to World Health Organization criteria (<130 g/L in men and <120 g/L in women).^10^ Anemia was common comorbidity in patients receiving PCI, and decreased baseline hemoglobin on admission was correlated with incrementally higher long‐term risk for ischemic stroke, mortality, and major bleeding events.^11^, ^12^, ^13^

Although the exact mechanism between anemia and MACE is not clear, patients with anemia tend to be older, have a variety of complications, and have poor basic status, which might be important reasons.^14^, ^15^ In addition, the underutilization of dual antiplatelets and statins might also be a potential cause of adverse clinical outcomes.^16^, ^17^ From a pathophysiological point of view, the necessary condition for coronary artery disease is an imbalance between cardiac oxygen supply and demand, and the presence of anemia further exacerbates the disequilibrium by decreasing oxygen‐carrying capacity while increasing oxygen consumption.^16^, ^18^ Furthermore, peripheral blood endothelial progenitor cells in acute coronary syndrome patients with anemia reduced in number and impaired function, indicating that the poor vascular healing capacity might also be involved as a part of the reason.^19^ Thirdly, aseptic inflammation mediated by immune response played an important role in coronary heart disease, including promoting atherosclerotic plaque formation and plaque instability,^20^ meanwhile anemia and inflammation interacted together to lead to unfavorable clinical events compared to patients without anemia.^21^, ^22^

This study further found that the incidence of MACE was lower in patients who had MI history, while the gender and age differences were not significant. This is not completely consistent with the cases we encountered in our actual clinical work and literature reports. The limited number of cases included in this study and regional differences may be one of the main reasons. In a follow‐up cohort study from Sappa et al., long‐term survival was lower in patients aged ≥85 years compared to younger patients.^23^ In a study that included 1111 STEMI patients undergoing reperfusion therapy, mortality in‐hospitalization was significantly higher in patients aged ≥65, even when anemia was not present.^7^ This also suggests that it is necessary to correct anemia in elderly patients who were performed PCI, which played an important role in improving their prognosis.

Admittedly, the study also has its limitations. First, the number of cases included in this cohort was relatively small, which might affect the statistical efficiency and required further analysis of large multi‐center samples. Second, this study was a secondary analysis based on previous studies, and the adjusted variables were limited, so there might be bias caused by other covariables. Finally, some patients with severe cardiovascular disease and other conditions were excluded from the study, so it was not clear whether the results applied to them.

## 5 CONCLUSIONS

In summary, for STEMI patients undergoing PCI, low preoperative hemoglobin was associated with high postoperative MACE incidence. Therefore, anemia should be corrected as far as possible especially in those with previous MI history to improve their prognosis.

## Data Availability

The dataset used during the study is available at https://doi.org/10.5061/dryad.pf56m.
